# Mind the gap—the use of sodium fluoresceine for resection of brain metastases to improve the resection rate

**DOI:** 10.1007/s00701-022-05417-1

**Published:** 2022-11-11

**Authors:** Johannes Kerschbaumer, Matthias Demetz, Aleksandrs Krigers, Daniel Pinggera, Antonio Spinello, Claudius Thomé, Christian F. Freyschlag

**Affiliations:** Department of Neurosurgery, Medical University of Innsbruck, Anichstr. 35, 6020 Innsbruck, Austria

**Keywords:** Brain metastases, Fluoresceine, Extent of resection, Neurosurgery, Neuro-oncology

## Abstract

**Introduction and purpose:**

Brain metastases appear to be well resectable due to dissectable tumor margins, but postoperative MRI quite often depicts residual tumor with potential influence on tumor control and overall survival. Therefore, we introduced sodium fluoresceine into the routine workflow for brain metastasis resection. The aim of this study was to evaluate whether the use of fluorescence-guided surgery has an impact on postoperative tumor volume and local recurrence.

**Material and methods:**

We retrospectively included patients who underwent surgical resection for intracranial metastases of systemic cancer between 11/2017 and 05/2021 at our institution. Tumor volumes were assessed pre- and postoperatively on T1-CE MRI. Clinical and epidemiological data as well as follow-up were gathered from our prospective database.

**Results:**

Seventy-nine patients (33 male, 46 female) were included in this study. Median preoperative tumor volume amounted to 11.7cm^3^ and fluoresceine was used in 53 patients (67%). Surgeons reported an estimated gross total resection (GTR) in 95% of the cases, while early postoperative MRI could confirm GTR in 72%. Patients resected using fluoresceine demonstrated significantly lower postoperative residual tumor volumes with a difference of 0.7cm^3^ (*p* = 0.044) and lower risk of local tumor recurrence (*p* = 0.033). The use of fluorescence did not influence the overall survival (OS). Postoperative radiotherapy resulted in a significantly longer OS (*p* = 0.001).

**Discussion:**

While GTR rates may be overrated, the use of intraoperative fluorescence may help neurosurgeons to achieve a more radical resection. Fluoresceine seems to facilitate surgical resection and increase the extent of resection thus reducing the risk for local recurrence.

## Introduction

Brain metastases (BM) are the most frequent intracranial neoplasms [3, 7, 35] and have a significant impact on the patients’ quality of life [8, 19, 37]. Considering the palliative treatment setting in the majority of patients with BM, timely, safe, and effective treatment is crucial [6, 37]. Various treatment options are available, depending on factors such as the size of the lesion, the number of BM, and the general condition of the patient.

Since the majority of chemotherapeutics do not overcome the blood–brain barrier and therefore do not perform well in the central nervous system (CNS) [38, 39], radiation therapy is the first choice in most patients [1, 40], Whole brain radiotherapy (WBRT) has been applied as palliative treatment, but showed unfavorable results in terms of overall survival (OS) and the patients’ cognitive outcome [2, 14, 25]. Thus, radiosurgical procedures representing a focal treatment gained more importance in recent years.

Surgical resection on the other hand is safely feasible, since BM are thought to be characterized by a well-established dissection plane between tumor and surrounding brain. Additionally, surgery has the advantage of rapid relief of the mass effect, can reduce the use of corticosteroids [13, 26], and leads to prolonged OS [24]. Resection is therefore used especially in larger BM with mass effect and in patients with a limited number of intracranial tumors. Also in oligometastatic disease of the brain, surgery may help by providing immediate symptom relief and reduction of tumor burden [11].

Overall, the incidence of BM is increasing due to improved systemic tumor control and an aging population [3]. Considering the palliative situation in most patients, it is crucial to maintain the highest possible quality of life (QoL) by resolving neurological deficits. Various intraoperative tools have been developed to maximize the extent of resection (EOR) while preserving neurologic function such as intraoperative monitoring, mapping, stimulation, and the use of fluorescence [16, 29, 31].

Surgical resection of BM is safe in the majority of cases, as postoperative outcome shows a low incidence of decline in the patients’ QoL [12, 32, 34]. However, the EOR is also relevant for the prognosis of BM, as complete tumor resection has been associated with prolonged survival [23]. The EOR on early postoperative MRI differs significantly from the expected EOR rated by the surgeons, as previously demonstrated [4, 23].

**Fig. 1:**
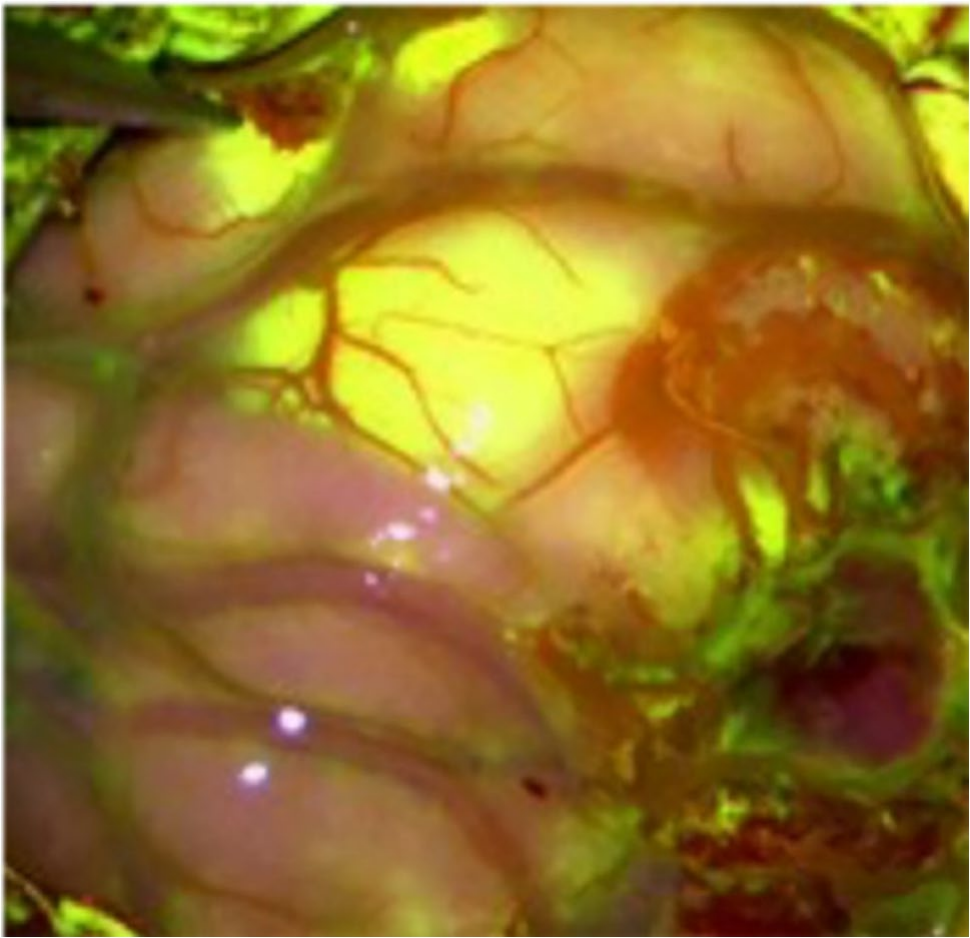
An example of sodium-fluoresceine with a clear delineation of the BM from surrounding tissue

Sodium fluoresceine (Na-Fl) has been introduced as a fluorescent dye in contrast enhancing (CE) lesions in the brain. It visualizes the tumor via staining of the disrupted blood–brain barrier and is applied in fluorescence-guided surgery for various brain tumors [9, 18]. Using a special filter (560 nm), the fluorescence can be visualized and can facilitate the resection, as the majority of BM present with contrast enhancement on preoperative MRI (Fig. 1). It has been reported that the use of fluoresceine can be used to increase the extent of resection with no increase in postoperative neurological damage, as recent study showed [9, 15, 22]. However, comparative data investigating the influence of fluoresceine on the discrepancy between expected EOR estimated by the surgeon and radiological EOR on postoperative MRI and on the residual tumor volume as well as on neurological postoperative outcome are limited.

The aim of this study was therefore to gain further understanding on the impact of fluoresceine on prognosis, postoperative outcome (neurological, radiological), and the impact on postoperative radiotherapy in patients undergoing surgical resection for a BM.

## Material and methods

All patients who underwent surgical resection of at least one BM at our department between November 2017 and May 2021 were retrospectively included from our prospective neuro-oncological database. Clinical and epidemiological data was gained from the electronic patients’ charts. Follow-up and postoperative neurological status as well as general oncological status using the Karnofsky Performance Score (KPS) were assessed retrospectively.

Additional parameters assessed from our database were primary tumor, number of brain metastases detected on preoperative MRI, the EOR determined on early (within 48 h) postoperative MRI, the neurosurgeons’ level of training (in training, fully qualified), the neurosurgeons’ intraoperatively estimated EOR as institutional standard during surgery, and cerebral as well as systemic progression on follow-up imaging according to RANO criteria [20].

All surgical interventions were performed using a Zeiss surgical microscope with a YELLOW 560 nm filter (Kinevo 900, Carl Zeiss Meditec, Oberkochen Germany).

Volumetric analysis was performed through segmentation using iPlan (BrainLab, München, Germany) on preoperative and early postoperative MRI. The postoperative MRI was performed within 48 h after surgery as standard of care at our institution for patients harboring malignant intracranial tumors.

Fluoresceine was used as a surgical adjunct following informed consent on the surgical procedure and the use of fluorescence. Application of fluoresceine was at the surgeons’ discretion and additionally influenced by availability of the appropriate surgical microscope with a 560-nm filter. Patients who underwent resection using fluoresceine were compared with patients with conventional resection without fluorescence. A subgroup analysis was performed, which only included patients with estimated GTR by the surgeon to investigate the role of fluoresceine on the extent of resection and the discrepancy between the surgeons’ estimation and the MRI-confirmed EOR.

### Statistics

Data processing and statistical evaluation was done in IBM SPSS Statistics (v.27.0 for Mac OS. Armonk, NY: IBM Corp.). In monovariate analysis, Mann–Whitney *U*-test for scale and ranked as well as chi-square for binominal variables were applied. The estimated OS was calculated in Kaplan–Meier processing and compared by log-rank test. Hazard ratios for overall survival were evaluated in Cox regression. Confidence interval and 1–alpha were set at 95%.

This retrospective analysis has been approved by the ethics committee of the Medical University of Innsbruck (1333/2021). The investigation was performed in accordance with the ethical standards of the 1975 Declaration of Helsinki and its amendments of 2013.

## Results

Seventy-nine patients (33 male, 46 female) with a median age of 63 years (interquartile range (IqR) 54.5–71.5) were included. Fluoresceine was used in 53 cases (67%). Median preoperative KPS amounted to 90 (“normal activity, minor signs of disease,” IqR 85–95). Tumor location and number of lesions are shown in Table 1.

**Table 1:** BM location: majority of BM presented with a supratentorial, non-eloquent location

	Number of patients	%
Singular BM	49	62
Multiple BM	30	38
Right hemisphere	40	51
Left hemisphere	39	49
Supratentorial	60	76
Infratentorial	19	24
Eloquent tumor location	18	23
Non-eloquent tumor location	61	77

Median preoperative tumor volume in T1-weighted contrast enhanced imaging was 11.7 cm^3^ (IqR 4.3–19.1). The surgeons reported an expected GTR in 95% of the cases. Early postoperative MRI, on the other hand, confirmed GTR only in 72% of the cases.

During follow-up, 21% of our patients developed local brain tumor progression after a mean of 10 months (standard deviation (SD) 7.3), while 36% showed a distant progression of brain tumors.

Sixty-six patients (84%) underwent radiotherapy after surgical resection. WBRT after surgery was applied in 26 (33%) patients. Half of the patients in this series were still alive at the time of statistical analysis. Patients who died during follow-up showed a median overall survival (OS) of 5 months (IqR 2–8).

Neither level of training (resident/consultant), tumor location, nor number of BMs had a significant influence (*p* > 0.05) on the use of fluoresceine in this study.

Patients who were resected using fluoresceine revealed significantly lower postoperative residual volumes (mean 0.23 cm^3^ using fluoresceine vs mean 0.54 cm^3^ without fluoresceine, *p* = 0.044). Accordingly, the local recurrence rate in the fluoresceine cohort was also significantly lower than in patients without fluoresceine use (*p* = 0.033). However, no impact on distant tumor progression could be shown (*p* > 0.05). Postoperative residual volume was not influenced by preoperative tumor volume, age, or training level of the surgeon (*p* > 0.05).

Estimated EOR and MRI-verified EOR are shown in Table 2, while Table 3 shows the differences in EOR on postoperative MRI between patients with and without fluorescein.

**Table 2:** Numbers and percentage of estimated and verified EOR. The previously reported discrepancy between surgeons’ estimated EOR and MRI-verified EOR could be confirmed in this study

	Fluoresceine	Percent	White light	Percent
Complete resection estimated	48	95%	22	96%
Incomplete resection estimated	3	5%	1	4%
Complete resection MRI	34	77%	12	60%
Incomplete resection MRI	10	23%	8	40%

**Table 3:** Patients who underwent fluoresceine-guided surgery showed a complete resection postoperatively in 83%. Patients who were resected without fluoresceine showed no postoperative residual tumor in only 64%

	Fluoresceine	Percent	White light	Percent
EOR < 60%	1	2%	0	0%
EOR 60–90%	1	2%	3	13%
EOR 90–99%	6	13%	5	23%
EOR 100%	40	83%	14	64%

Patients who underwent fluoresceine-guided resection not only demonstrated significantly lower postoperative residual tumor volumes and a higher extent of resection, but also a lower discrepancy between the surgeons’ estimated EOR and the MRI-verified EOR (*p* = 0.05). Fluoresceine use had no significant influence on OS (*p* > 0.05) in the Cox regression analysis.

Postoperative radiotherapy (RTX), however, could be shown as an important factor to prolong OS significantly with a mean of 16.1 months (SD 7.4) in patients undergoing radiotherapy compared to 6.4 months (SD 2.1) in patients without RTX (*p* = 0.001).

Gender also influenced the OS significantly (*p* = 0.005), with male patients demonstrating a worse outcome than female patients. Neither the primary tumor, preoperative general condition (KPS), nor tumor volume influenced the prognosis of patients with regard to OS in this study. We could not show any differences in sodium fluoresceine visualization between metastases of different primary tumors in this study.

## Discussion

The use of intraoperative sodium fluoresceine in the resection of brain metastases significantly reduced residual tumor volume and significantly decreased the local recurrence rate. Furthermore, fluoresceine could help to reduce the gap between estimated EOR and MRI-confirmed EOR obviously by providing a better intraoperative tumor delineation. As expected, there was no effect of fluoresceine use on distant tumor recurrence, emphasizing the importance of postoperative adjuvant treatments like radiotherapy. In this study, radiotherapy demonstrated a major impact on the overall survival of the patients. Tumor volume and preoperative general status (KPS) showed no effect on overall survival, indicating that even patients with a large lesion and poor preoperative status may benefit from resection.

Brain metastases often occur in late-stage oncologic disease and can severely compromise patient outcome and QoL [10, 33, 36]. Surgical resection is the treatment of choice for lesions with mass effect [5, 27]. However, maintaining the highest possible QoL of patients in a palliative situation is of utmost importance. The use of fluoresceine can be helpful, as improved visibility of the MRI CE tumors can be achieved [9]. However, data on the use of fluoresceine in the clinical setting are limited. This intraoperative adjunct has been used regularly at our department since 2017, but use was deliberately limited by logistic and other factors, thus prompting this analysis.

Residual postoperative tumor volume was significantly lower in this study when fluoresceine was used. This could be due to a better intraoperative delineation of the tumor margins, consequently increasing the EOR. Despite a usually detectable intraoperative BM dissection plane, the EOR estimated by the surgeons noticeably differs from the EOR on early postoperative MRI, as reported previously [4, 23] and confirmed in our cohort. Fluoresceine seems to be a helpful tool to minimize damage to surrounding brain tissue and may even be useful in metastases in eloquent regions in order to preserve functional areas [30]. Nevertheless, this has to be properly evaluated as a more extensive resection may potentially endanger neighboring eloquent tissue even in BM.

The expected better intraoperative depiction of tumor margins may increase the EOR by a more extensive resection following the fluorescence. Patients operated with fluoresceine also showed a lower discrepancy between the surgeon’s estimated EOR and the MRI-confirmed EOR. Indeed, this has clinical relevance, as EOR is often overestimated in patients with BM, as this has been previously reported and could be confirmed in our investigation.

The use of fluoresceine not only resulted in significantly lower tumor residual volumes, but importantly also in significantly reduced local tumor recurrence. Since BM and their recurrence can remarkably decrease the patients’ prognosis and QoL, fluoresceine use could entail a better outcome [19]. As expected, distant brain tumor and systemic oncologic progression was not influenced by the use of fluoresceine. This underlines the need of effective adjuvant treatments like RTX and systemic anti-cancer agents to achieve the best possible outcome in terms of OS and QoL. Postoperative RTX, as previously mentioned, played a key role concerning OS in this study. Patients without RTX exhibited a significantly worse prognosis and shortened outcome, as reported in literature [17, 21]. Therefore, RTX should be performed whenever possible. The worse prognosis of male patients in malignant disease has also been known and could be confirmed in this series [28].

On the other hand, preoperative tumor volume and KPS had no significant impact on OS in our study. We conclude that patients with a possibly BM-related poor general condition may also benefit from surgical treatment. Moreover, even large lesions seem to be well resectable with fluoresceine.

### Limitations

The study is limited by its retrospective and monocentric design. Furthermore, no randomization was conducted and the decision whether to use fluoresceine or not was based solely on the discretion of the surgeon and the availability of the appropriate microscope.

## Conclusion

The intraoperative use of sodium fluoresceine resulted in our cohort in significantly lower postoperative residual tumor volumes and significantly lower incidences of local tumor recurrence. Thus, fluorsceine might enhance the surgical resection by reducing the discrepancy between the surgeons’ estimated and the radiologically verified EOR. However, patients’ OS was strongly influenced by postoperative RTX and not by the use of fluorsceine, which highlights the importance of multidisciplinary treatment to achieve the best possible outcome for OS and QoL of BM patients.

## Data Availability

The datasets generated during and/or analyzed during the current study are available from the corresponding author on reasonable request due to privacy and ethical restrictions.
